# Capturing biodiversity: linking a cyanobacteria culture collection to the “scratchpads” virtual research environment enhances biodiversity knowledge

**DOI:** 10.3897/BDJ.4.e7965

**Published:** 2016-04-06

**Authors:** Spyros Gkelis, Manthos Panou

**Affiliations:** ‡ Department of Botany, School of Biology, Aristotle University of Thessaloniki, Thessaloniki, Greece

**Keywords:** cyanobacteria, database, Scratchpads, taxonomy, morphology, phylogeny, biodiversity informatics

## Abstract

**Background:**

Currently, cyanobacterial diversity is examined using a polyphasic approach by assessing morphological and molecular data (Komárek 2015). However, the comparison of morphological and genetic data is sometimes hindered by the lack of cultures of several cyanobacterial morphospecies and inadequate morphological data of sequenced strains (Rajaniemi et al. 2005). Furthermore, in order to evaluate the phenotypic plasticity within defined taxa, the variability observed in cultures has to be compared to the range in natural variation (Komárek and Mareš 2012). Thus, new tools are needed to aggregate, link and process data in a meaningful way, in order to properly study and understand cyanodiversity.

**New information:**

An online database on cyanobacteria has been created, namely the Cyanobacteria culture collection (CCC) (http://cyanobacteria.myspecies.info/) using as case studies cyanobacterial strains isolated from lakes of Greece, which are part of the AUTH culture collection (School of Biology, Aristotle University of Thessaloniki). The database hosts, for the first time, information and data such as morphology/morphometry, biogeography, phylogeny, microphotographs, distribution maps, toxicology and biochemical traits of the strains. All this data are structured managed, and presented online and are publicly accessible with a recently developed tool, namely “Scratchpads”, a taxon-centric virtual research environment allowing browsing the taxonomic classification and retrieving various kinds of relevant information for each taxon.

## Introduction

Biodiversity is the study of the variety of life at all possible levels of the biological organisation (from genes to ecosystems) and scales of observation (from local to global). Therefore, studies of biodiversity are predicated on the capacity to bring together information from across a diverse spectrum of scientific fields (Koureas et al. 2016). The Mediterranean area is a known biodiversity hot spot, however, diversity of microbes is substantially underestimated or unexplored (Coll et al. 2010). The diversity of freshwater cyanobacteria, especially those involved in water blooms, has been brought into attention as studies have shown that prolonged cyanobacterial blooms, dominated by known toxic species, can occur (Gkelis et al. 2014). Furthermore, cyanobacteria are a prolific source of natural products, known from just a handful of genera (Dittmann et al. 2015) and emerging data are providing a genetic basis to the natural product diversity. This is expected to set up an integrated research workflow that will increase the efficiency of biodiscovery pipelines.

Cyanobacteria are a large and morphologically very diverse group of photosynthetic prokaryotes, which occur almost in every illuminated habitat, and quantitatively are among the most important organisms on Earth (Whitton 2012). Today, cyanobacterial diversity is examined using a polyphasic approach by assessing morphological and molecular data (Komárek 2015). The comparison of morphological and genetic data is sometimes hindered by the lack of cultures of several cyanobacterial morphospecies and inadequate morphological data of sequenced strains (Rajaniemi et al. 2005). Furthermore, in order to evaluate the phenotypic plasticity within defined taxa, the variability observed in cultures has to be compared to the range in natural variation (Komárek and Mareš 2012).

Biodiversity research is at a pivotal point with research projects generating data at an ever increasing rate. Structuring, aggregating, linking and processing these data in a meaningful way is a major challenge (Koureas et al. 2016). The need for efficient informatics tools in biodiversity research is constantly increasing, and this is reflected in the volume of different biodiversity information projects (>680) (http://www.tdwg.org/biodiv-projects/) currently running at a local, regional or global level. However, only very few (less than five) projects are dedicated to bacteria or algae. To the best of our knowledge, apart from the AlgaeBase (Guiry and Guiry 2016) comprising information on all terrestrial, marine and freshwater algae, there is only one online database listing cyanobacteria genera (Komárek and Hauer 2013); other databases contain only taxonomic information and/or images.

In this paper, we present “Cyanobacteria culture collection” a database on cyanobacteria hosting, for the first time, information such as morphology/morphometry, biogeography, phylogeny, microphotographs, distribution maps, toxicology, and biochemical traits of cyanobacteria strains isolated from freshwaters of Greece. All those data are structured managed, and presented online and are publicly available through Scratchpads (Smith et al. 2009).

## General description

### Purpose

The purpose of this database is to make available data associated with cyanobacteria in Greece. The database features information about different traits (morphological, morphometric, biochemical) for cyanobacteria strains. The dataset represents a long-term and ongoing survey that aims to be useful in future investigations of cyanobacteria diversity, phylogeny, ecology, new metabolites discovery.

## Sampling methods

### Study extent

This dataset is primarily developed to sum our ongoing effort on exploring the biodiversity (morphological, genetic, metabolite) of photosynthetic organisms. Thus, the strains comprising the dataset are from freshwaters of Greece isolated during the past 15 years. However, marine cyanobacteria strains isolated from the Aegean Sea and thermophilic strains isolated from thermal springs (unpublished data) are soon to be included.

### Sampling description

The strains were isolated during the years 1999-2015 from 12 different freshwater lakes and reservoirs (Table 1). Strains were isolated on solid and/or liquid growth media using classical microbiological techniques and grown as batch clonal unialgal cultures; all strains were derived from a single colony or trichome. More information on sampling sites and strain isolation are given in Gkelis et al. (2015).

### Quality control

The isolates are deposited in Aristotle University of Thessaloniki (AUTH) microalgae collection (Department of Botany, School of Biology). A Zeiss Axio imager z2 (Carl Zeiss, Germany) microscope using bright field and differential interference contrast (EC Plan-Neofluar 5x/0,16,EC Plan-Neofluar 10x/0.3, Plan- Apochromat 20x/0.8, Plan-Neofluar 40x/0.75 DIC, Plan- Neofluar 63x/1.25 Oil DIC, Plan-Neofluar 100x/1.30 Oil DIC) was used to assess morphological and morphometric characters. Microphotographs used in the database were taken with an Axio Cam MRc5 digital camera (Carl Zeiss, Germany). The strains were identified to the species or genus level according to Komárek and Anagnostidis (1999), Komárek and Anagnostidis. (2005), Komárek (2013), taking into consideration the current taxonomic status (Komárek 2015).

## Geographic coverage

### Description

All taxa in the database were isolated from several Greek freshwater bodies. However, the database is constantly being expanded, so strains from other locations across Greece will be present in the database in the near future.

### Coordinates

38°27'N and 41°11'N Latitude; 20°51'E and 23°21'E Longitude.

## Taxonomic coverage

### Description

**Figure 1.**
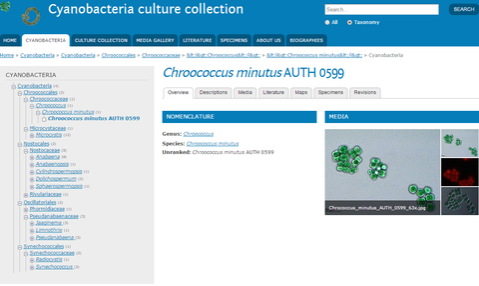
Preview of the “Cyanobacteria culture collection” database collection. The Taxonomy system is presented as part of the "Cyanobacteria" tab; an overview of the strain *Chococcus minutus* AUTH 0599 is shown as an example.

At present, the database contains 49 strains, representing 22 taxa, 16 genera and seven families (Chroococcaceae, Microcystaceae, Hapalosiphonaceae, Nostocaceae, Rivulariaceae, Phormidiaceae, Pseudanabaenaceae, Synechococcaceae), belonging to four orders in Cyanobacteria class: Chroococcales, Nostocales, Oscillatoriales and Synechococcales. A total of 18 taxa belong to Chroococcales, 15 to Nostocales, 12 to Oscillatoriales and four taxa the Synechococcales (Table 1). The taxonomy of the strains is shown online by clicking the tab "Cyanobacteria" (Fig. 1).

### Taxa included

**Table:** 

Rank	Scientific Name	
species	Chroococcus minutus	
species	Microcystis flos-aquae	
species	Microcystis aeruginosa	
genus	Synechococcus	
species	Limnothrix redekei	
genus	Jaaginema	
genus	Pseudanabaena	
genus	Anabaena	
genus	Dolichospermum	
genus	Calothrix	
species	Planktothrix agardhii	
species	Pseudanabaena limnetica	
genus	Radiocystis	
species	Sphaerospermopsis aphanizomenoides	
species	Cylindrospermopsis raciborskii	
genus	Hapalosiphon	
species	Cuspidothrix elenkinii	

## Traits coverage

**Figure 2.**
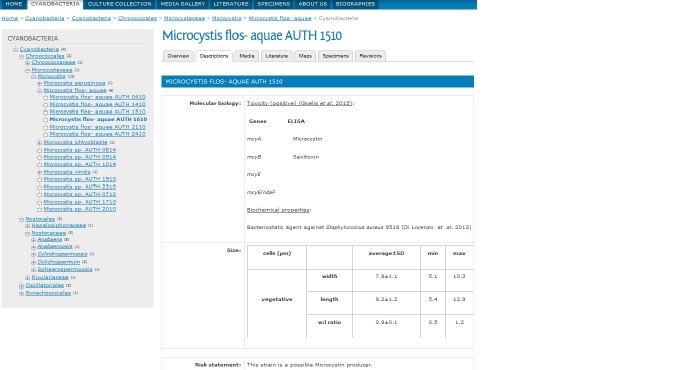
The "Descriptions" tab including morphometric (cell's width, filament's length), toxicity and biochemical traits data for the strain *Microcystis flos-aquae* AUTH 1510. These data are shown after clicking the desirable taxon in the backbone taxonomy.

**Figure 3.**
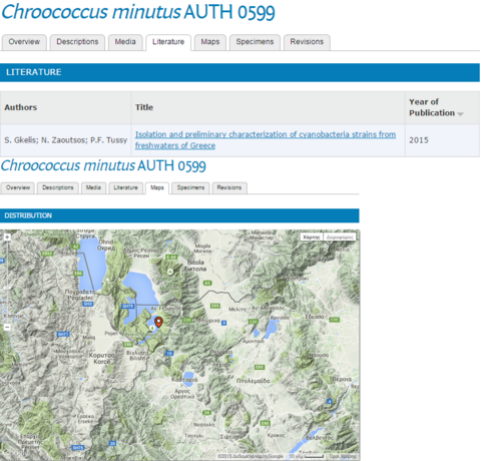
“Literature” and ”Maps” tabs for the strain *Chroococcus minutus* AUTH 0599.

Information for each strain are given in different tabs after choosing a particular strain. Some strains were characterised based on their morphological features and 16S rRNA gene sequences (Gkelis et al. 2005), screened with respect to their ability to produce cyanotoxins (Gkelis et al. 2015) or their antibacterial traits (Lorenzo et al. 2013). This information is contained in the "Descriptions" tab where all available morphological/morphometrical, toxicity and biochemical data, are given (Fig. 2). The ”Media” tab contain microphotographs, whereas "Literature" and "Maps" refer to the relevant literature and the region where the strain was isolated, respectively (Fig. 3). About 12 traits per isolate are currently given in the database.

## Collection data

### Collection name

Aristotle University of Thessaloniki (AUTH) microalgae collection (Department of Botany, School of Biology)

### Collection identifier

AUTH

### Specimen preservation method

Living Specimens

### Curatorial unit

The isolates are maintained in Aristotle University of Thessaloniki (AUTH) microalgae collection (Department of Botany, School of Biology). Cultures are grown as liquid batch cultures at 20±2 oC or (25±1 oC for Microcystis) at a photosynthetic photon flux density of 20 μmol m-2 s-1 provided by cool white light fluorescent lamps (Sylvania Standard F36W/154-T8, SLI) in a 16:8 h light:dark cycle.

## Usage rights

### Use license

Open Data Commons Attribution License

### IP rights notes

This work is licensed under a Creative Commons Attribution-NonCommercial-ShareAlike 4.0 International License.

## Data resources

### Data package title

Cyanobacteria culture collection

### Resource link

http://cyanobacteria.myspecies.info/

### Number of data sets

1

### Data set 1.

#### Data set name

Cyanobacteria culture collection

#### Number of columns

9

#### Download URL

http://cyanobacteria.myspecies.info/specimen_observation

#### 

**Data set 1.** 

Column label	Column description
Basis of record	Living specimen or preserved sample
Catalogue number	The number of each strain in the culture collection
Collection code	The code of each strain in the culture collection
Institution code	The institution's code for the collection
Taxonomic name	Taxonomic name of each strain
Date collected	Sample collection date
GenBank number(s)	The number(s) for strains' sequences, where available
Location	The waterbody where each strain was isolated from
Date identified	The date each strain was identified

## Figures and Tables

**Table 1.** Cyanobacteria strains included in the database and their origin.

**Strain**	**Origin** **(Lake or Reservoir)**	**Collection date**
*Chroococcus minutus* AUTH 0599	Mikri Prespa	5/8/1999
*Microcystis flos-aquae* AUTH 0410	Pamvotis	21/8/2010
*Microcystis aeruginosa* AUTH 0610	Kastoria	24/8/2010
*Microcystis* sp. AUTH 0710	Kastoria	24/8/2010
*Microcystis flos-aquae* AUTH 1410	Pamvotis	1/11/2010
*Microcystis flos-aquae* AUTH 1510	Pamvotis	1/11/2010
*Microcystis* sp. AUTH 1610	Pamvotis	1/11/2010
*Microcystis* sp. AUTH 1710	Pamvotis	1/11/2010
*Microcystis viridis* AUTH 1810	Pamvotis	1/11/2010
*Microcystis* sp. AUTH 2010	Pamvotis	1/11/2010
*Microcystis* sp. AUTH 2110	Pamvotis	1/11/2010
*Microcystis* sp. AUTH 2310	Pamvotis	1/11/2010
*Microcystis flos-aquae* AUTH 2410	Pamvotis	1/11/2010
*Synechococcus* sp. AUTH 0499	Cheimaditis	5/8/1999
*Synechococcus* sp. AUTH 3010	Pamvotis	1/11/2010
*Limnothrix redekei* AUTH 0310	Doirani	21/8/2010
*Jaaginema* sp. AUTH 0110	Volvi	21/8/2010
*Jaaginema* sp. AUTH 0210	Doirani	21/8/2010
*Jaaginema* sp. AUTH 2210	Kerkini	21/8/2010
*Pseudanabaena* sp. AUTH 0104	Pikrolimni	27/9/2004
*Anabaena* cf. *oscillarioides* AUTH 0199	Paralimni	19/7/1999
*Anabaena* sp. AUTH 0299	Paralimni	19/7/1999
*Anabaena* cf. *cylindrica* AUTH 0699	Amvrakia	19/8/1999
*Anabaena* sp. AUTH 0799	Kerkini	26/8/1999
*Anabaena* sp. AUTH 0899	Kerkini	26/8/1999
*Anabaena* sp. AUTH 2510	Doirani	21/8/2010
*Anabaena* sp. AUTH 2610	Doirani	21/8/2010
*Anabaena* sp. AUTH 2710	Doirani	21/8/2010
*Calothrix* sp. AUTH 0399	Pamvotis	22/7/1999
*Limnothrix redekei* AUTH 0114	Karla	11/09/2013
*Limnothrix redekei* AUTH 0214	Karla	11/09/2013
*Limnothrix redekei* AUTH 0314	Karla	11/09/2013
*Anabaenopsis elenkinii* AUTH 0414	Karla	11/09/2013
*Planktothrix agarhii* AUTH 0514	Karla	11/09/2013
*Pseudanabaena limnetica* AUTH 0614	Karla	11/09/2013
*Pseudanabaena mucicola* AUTH 0714	Karla	11/09/2013
*Microcystis* AUTH 0814	Karla	11/09/2013
*Microcystis* AUTH 0914	Karla	11/09/2013
*Microcystis* AUTH 1014	Karla	11/09/2013
*Synechococcus* AUTH 1114	Karla	11/09/2013
*Radiocystis* AUTH 1214	Karla	11/09/2013
*Sphaerospermopsis aphanizomenoides* AUTH 1314	Kalamaki	22/11/2013
*Cylindrospermopsis raciborskii* AUTH 1414	Kalamaki	22/11/2013
*Limnothrix redekei* AUTH 1514	Kalamaki	22/11/2013
*Anabaena*/*Dolichospermum* AUTH 1614	Kalamaki	21/02/2014
*Anabaena*/*Dolichospermum* AUTH 1714	Kalamaki	21/02/2014
*Hapalosiphon* sp. AUTH 0115	Trichonida	08/01/2015
